# Probing the role of stochasticity in a model of the embryonic stem cell – heterogeneous gene expression and reprogramming efficiency

**DOI:** 10.1186/1752-0509-6-98

**Published:** 2012-08-13

**Authors:** Vijay Chickarmane, Victor Olariu, Carsten Peterson

**Affiliations:** 1 Computational Biology & Biological Physics, Lund University, Lund, Sweden; 2 Division of Biology, California Institute of Technology, , Pasadena, USA; 3 Lund Strategic Research Center for Stem Cell Biology and Cell Therapy, Lund University, Lund, Sweden

**Keywords:** Stem cells, Heterogeneity, Stochasticity, Computational model, Differentiation, Reprogramming

## Abstract

**Background:**

Embryonic stem cells (ESC) have the capacity to self-renew and remain pluripotent, while continuously providing a source of a variety of differentiated cell types. Understanding what governs these properties at the molecular level is crucial for stem cell biology and its application to regenerative medicine. Of particular relevance is to elucidate those molecular interactions which govern the reprogramming of somatic cells into ESC. A computational approach can be used as a framework to explore the dynamics of a simplified network of the ESC with the aim to understand how stem cells differentiate and also how they can be reprogrammed from somatic cells.

**Results:**

We propose a computational model of the embryonic stem cell network, in which a core set of transcription factors (TFs) interact with each other and are induced by external factors. A stochastic treatment of the network dynamics suggests that NANOG heterogeneity is the deciding factor for the stem cell fate. In particular, our results show that the decision of staying in the ground state or commitment to a differentiated state is fundamentally stochastic, and can be modulated by the addition of external factors (2i/3i media), which have the effect of reducing fluctuations in NANOG expression. Our model also hosts reprogramming of a committed cell into an ESC by over-expressing OCT4. In this context, we recapitulate the important experimental result that reprogramming efficiency peaks when OCT4 is over-expressed within a specific range of values.

**Conclusions:**

We have demonstrated how a stochastic computational model based upon a simplified network of TFs in ESCs can elucidate several key observed dynamical features. It accounts for (i) the observed heterogeneity of key regulators, (ii) characterizes the ESC under certain external stimuli conditions and (iii) describes the occurrence of transitions from the ESC to the differentiated state. Furthermore, the model (iv) provides a framework for reprogramming from somatic cells and conveys an understanding of reprogramming efficiency as a function of OCT4 over-expression.

## Background

Understanding the molecular networks which give rise to pluripotency in embryonic stem (ES) cells is crucial for among other things developing reprogramming strategies. Recent work has shed light on several key aspects of the underlying network and its interaction with external factors, in particular the chemical media which maintain the cells [1]. The current understanding is that ESCs occupy a multiplicity of sub-states, with stochastic transitions between them. One aim is to understand the molecular interactions that maintain cells in a pluripotent state, destabilize this state leading to commitment, as well as allow a return to the pluripotent state from a committed state. Given the substantial experimental efforts currently underway to understand these mechanisms, a computational systems biology approach seems like a way forward within which such questions could be formulated [2-6]. As in many other biomedical problem areas, a computational approach would here allow diverse experimental results to be absorbed into the formulation of the model, but more importantly, could serve as a hypothesis generator to test mechanisms through further experimentation. The recognition that states of a ES cell are read out by the gene expression of key regulators, has lead to a simple hypothesis regarding the pluripotent nature of the ESC [7]. An ES cell can be in a “ground state”, in which it is neutral to any developmental specification. However, it is possible for the cell to transition to a differentiated state. Here we explore the dynamics of a simplified network model representing key elements of ESC transcription factor and signaling regulators to suggest mechanisms for such a transition state picture.

At the heart of the pluripotency network lies the triad OCT4, SOX2 and NANOG [8-10], where OCT4 and SOX2 act together as a heterodimer regulating several genes including NANOG, OCT4 and SOX2 [11]. There are additional TFs that also impact pluripotency. The exact regulatory mechanisms in the network with impact on pluripotency remain to be fully understood. However, it appears that self-reinforcing mechanisms through feedback of these key regulators upon themselves seem to be instrumental. Interacting with these key components in mice are external factors like Leukemia Inhibiting Factor (LIF), which can substitute for feeders by activating the transcription factor STAT3 that inhibits ES differentiation [12,13]. Another factor, Bone Morphogenetic Protein (BMP4), has been shown to inhibit the differentiation proteins and thus can be used as a replacement for serum [14]. There are corresponding factors active in humans. The common media for maintaining stem cells in cultures is LIF plus serum or BMP4. It has been shown that serum/BMP4 can be replaced by small molecules which inhibit FGF4 receptor tyrosine kinases and the ERK cascade (2i/3i medium) [15]. The 2i/3i (two or three types of differentiation inhibiting molecules) medium is used successfully to maintain stem cells *in vitro* in combination with or without LIF.

Biochemical systems naturally exhibit stochastic fluctuations due to random interaction processes, gene transcription and translation as well as degradation. Recent studies have explored the role of stochastic fluctuations in a variety of organisms ranging from bacteria to mammalian cells [16,17]. In ESCs, it was shown that the expression of some transcription factors important for pluripotency are heterogeneous when cells are maintained in the “classical” environment i.e. LIF plus BMP4 or serum. Stochasticity or heterogeneity has been observed in key stem cell TFs such as NANOG [18-20], REX1 [21], STELLA [22]. Based upon these observations, it appears that stem cells exist in a multitude of sub-states, where each sub-state represents a certain multi-distribution of TF concentrations. In particular, NANOG shows more heterogeneity than OCT4 and SOX2 [18,20]. Cells expressing lower levels of NANOG are more prone to differentiate [18,23], thereby conferring a stochastic component to the ability of the cell to self-renew. Hence, the state space of ESCs is intricately woven into the heterogeneous gene expression of some of the key regulators of the network.

Underlying the ability of NANOG to act as a “gatekeeper” of pluripotency [24], is the fact that OCT4-SOX2 also induces FGF4, a differentiation promoting growth factor [7]. The ES cell requires OCT4 and SOX2 to maintain it in a pluripotent state, while at the same time pushing it towards differentiation. NANOG is thought to prevent differentiation, and hence when it reaches low levels, the probability to commit increases. How FGF4 fits into this network has so far not been computationally explored. Mouse ESCs can be maintained in a pluripotent state, through introduction of small molecule inhibitors. Ying et al. [15] discovered two different sets of small molecule inhibitors; 3i – FGF receptor inhibitor, Mitigen activated protein (MAP) kinase/ERK kinase - MEK inhibitor and GSK3 inhibitor, 2i – MEK inhibitor and a GSK3 inhibitor. Wray et al. [25] established that the expressions of NANOG and REX1 within the mouse ES cultures under 2i conditions were not heterogeneous i.e. only NANOG high or REX high are present, suggesting the existence of cells in a state that is intrinsically less fluctuating. This could be denoted a true “ground” state, which they suggested is an inherent stable pluripotency network governed by OCT4, SOX2 and NANOG, but, which is perturbed by Erk signaling acting through the FGF receptors.

It follows that a quantitative analysis of network dynamics could improve our understanding of the multiple states of the ESC. Previous purely deterministic studies have explored the dynamics of the OCT4-SOX2-NANOG regulatory network, as well as its role in determining the cell fate, i.e the final lineage: epiblast, trophectoderm and endoderm [26,27]. However, neither of these computational studies analyzed heterogeneity in NANOG expression. Kalmar et al. [20] suggested by stochastic modeling of a simplified stem cell network based upon observations, how NANOG fluctuations could make the stem cell state transition between multiple states. Their model involved feedbacks, both positive and negative between OCT4 and NANOG which lead to NANOG levels cycling between high and low levels as an excitable system. Subsequently Glauche et al. [28] further studied the nature of such stochastic transitions with two different model scenarios. In one model NANOG, which is induced by OCT4-SOX2 can act as a bistable switch, and can transition between high and low levels. In the other model, which is based upon an activator-repressor mechanism, NANOG can oscillate on a fixed limit cycle, and can recapitulate the observed heterogeneity in NANOG levels. Hence, several types of mechanisms could lead to NANOG heterogeneity. It is also suggested how NANOG can act as a gatekeeper by suppressing any differentiation signals which would ultimately make the cell transition into a differentiated cell. However, in [28], the signal to differentiate is external, and cells therefore cannot differentiate spontaneously as observed.

In this work we build upon these ideas by further analyzing how fluctuations in NANOG play a role in both allowing cells to transition between ES sub-states and then to finally exit irreversibly into a differentiated state. However, this occurs in a spontaneous fashion. Key to our approach, which is different from that of refs. [20,28], is the development of a self-organized network, in which the pluripotent network governed primarily by OCT4-SOX2-NANOG interacts with a differentiation pathway gene denoted by “G”. Candidates for G are for example GATA6 and SOX17. It is the stochastic dynamics of this network in which several types of feedbacks give rise to the observed stochastic stem cell fate. The noise therefore is internal to the network, with external stimuli controlling the strength of the fluctuations. Hence, stem cells can spontaneously change fate in accordance with observations. This also allows us to answer the second question as to how reprogramming can be simulated in our model. In [29] it has been shown that over-expression of OCT4 can lead to reprogramming a somatic cell to an ESC. However, the efficiency is maximal for the levels of OCT4 within a certain window [30]. Our model can reproduce this result, and we show how the interaction between OCT4, NANOG and the differentiation pathway gene G lead to this result.

## Results and discussion

### A simplified computational model of the ESC

Our simplified ESC network model considers a combination of positive and negative feedbacks between OCT4-SOX2 and NANOG and G. With stochastic simulations we demonstrate the permissive nature of this self-contained network – most cells retain pluripotency except for a fraction that get pushed towards differentiation. This model, is based upon an epigenetic effect by which OCT4 regulates NANOG, is also employed for reprogramming somatic cells into ESC.

**Figure 1:**
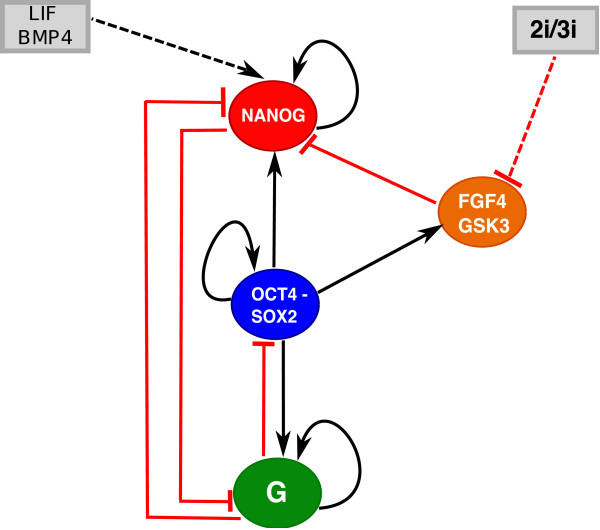
**The transcription factor interaction circuit along with external factors influences.** The core network for the mutual and self-regulatory interactions between NANOG, OCT4-SOX2 heterodimer, FGF4 and differentiation gene G. The dashed lines indicate the effect of external factors when cells are maintained in two different media, LIF+BMP4 and the 2i/3i respectively. The dynamical model is based upon the following (see Methods): OCT4-SOX2 induces NANOG. NANOG dimerizes and regulates itself positively. NANOG represses G, which regulates itself positively. OCT4-SOX2 induces G and the latter suppresses both NANOG and OCT4-SOX2. LIF induces NANOG through Klf4. OCT4-SOX2 induces FGF4, which suppresses NANOG. The 2i/3i medium suppresses FGF4.

The heterodimer OCT4-SOX2 is known to serve as an activator of OCT4, SOX2 and NANOG [11]. As in [28], we simplify the interaction of OCT4 and SOX2 with NANOG as shown in Figure 1. The feedback between NANOG and OCT4-SOX2 must be weak. Otherwise it would be inconsistent with low levels of NANOG and high levels of OCT4 and SOX2 as pointed out in [20,28]. Hence, we do not explicitly have NANOG inducing OCT4 and SOX2 in contrast to refs. [26,27]. To describe both the embryonic as well as the differentiated state, we include G in the circuit. One candidate for G is Sox17, which was shown to play a role in the control of differentiation of ESCs into extra embryonic endoderm [31]. SOX17 interferes with the self-renewal program by inhibiting SOX2, OCT4 and NANOG [31]. Another candidate for G is GATA6, which is responsible for endoderm formation and also mutually antagonizes NANOG [27]. In [28], the authors assumed an external signal promoting differentiation. However, in our approach the gene G is regulated by the ESC circuit itself, and hence is part of the network which determines the cell fate. Our circuit also includes the differentiation promoting autocrine growth factor FGF4, which is shown schematically in Figure 1 to repress NANOG [32-34]. It has been suggested that FGF4 acts upstream in the induction of differentiation [32,35]. The Fgf4 gene is expressed in mouse embryonic stem cells and only OCT-SOX complexes are able to promote its transcriptional activation [36]. Inhibition of FGF4 along with GSK3 consolidates the ESC self-renewal and pluripotency [15,25]. As in [27], we have assume mutual antagonism between NANOG and the differentiation gene G, as well as activation of G by OCT4-SOX2. As shown in [27], over-expression of OCT4 could either lead to the establishment or loss of the stem cell fate, depending on the level of OCT4. We have also considered epigenetic regulation of NANOG as it has been suggested that OCT4 activates the histone demethylases Jmjd2c, which in turn exposes key pluripotent genes to regulation, among them NANOG [37]. Including this mechanism in the model results in sharpening the non-linearity of the OCT4-NANOG interaction.

### Exploring the ground state of the ESC

#### Commitment – transition from the stem cell state to a differentiated state

We first compute the steady states of the system for different values of LIF using the deterministic rate equations (Eq. 1) for the circuit in Figure 1 with the parameters given in Table 1.

**Table 1:** **Parameters values used in Figures **3** and **4

**Parameters**																
***k***_**0**_	***c***_**0**_	***c***_**1**_	***c***_**2**_	***c***_**3**_	***c***_**4**_	***e***_**0**_	***e***_**1**_	***e***_**2**_	***a***_**0**_	***a***_**1**_	***a***_**2**_	***b***_**0**_	***b***_**1**_	***b***_**2**_	***b***_**3**_	***γ***
0.005	0.01	0.4	1	0.1	0.00135	0.01	1	1	0.01	1	5	0.005	0.005	1	1	0.01

With dynamics resulting from the interactions between G, NANOG and OCT4-SOX2, there are basically two states of the system: (i) the stem cell state, when OCT4-SOX2 and NANOG are ON and G is OFF, and (ii) vice versa for the somatic state.

In the somatic state G is high and both OCT4-SOX2 and NANOG are suppressed and hence OFF. This state remains even when increasing LIF since the model for the NANOG gene regulatory function is based upon a simplified epigenetic mechanism. For Nanog to be activated, the Nanog promoter must be bound by OCT4 along with any of its activators OCT4, NANOG (autoregulation), LIF. To be repressed, Nanog must be bound by OCT4 along with its repressors FGF4 and G.

Adding LIF, has no effect on NANOG if OCT4 is OFF, since LIF cannot access NANOG. However, if initially the cell is in a stem cell state with high OCT4-SOX2, then OCT4-SOX2 exposes NANOG, which allows LIF to induce NANOG. This in turn leads to suppression of G, which finally relieves the suppression on OCT4-SOX2. These sequential negative interactions implement a positive feedback loop between NANOG and OCT4-SOX2 (analogous to a different mechanism suggested in [28]). Additional file 1: Figure S1A displays the two states of the cell. The regulation of NANOG occurs through a feed-forward loop [38], in which OCT4 directly activates NANOG and indirectly represses NANOG through FGF4. Additional file 1: Figure S1B shows that adding 2i/3i to the media leads to suppression of FGF4 (magenta), and hence relieves NANOG from repression.

So far we have described a deterministic approach. However, chemical reactions are necessarily stochastic, and hence protein levels fluctuate in time [39-41]. We assume that all of the stochasticity originates from within the network, i.e internal noise, as it is entirely due to random events of protein production and degradation for each of the molecular components with no external noise [42]. Since this noise is generated by the network itself, it could be regarded as “permissive”, which has been conjectured to be the source of hematopoietic commitment [43,44]. To study the effects of stochasticity, we used a Gillespie approach where the deterministic equations (see Methods) provide transition rates for a master equation. The latter is simulated by a Monte Carlo procedure to provide the time evolution of the concentration levels [45].

#### Stochastic dynamics under LIF conditions

**Figure 2:**
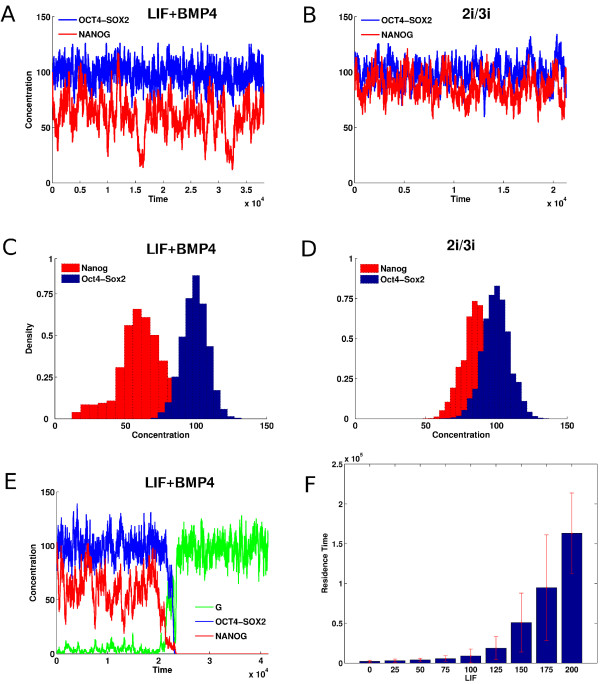
**Time series and distributions of [OS], [N] and [G] concentrations for the stochastic dynamics of the gene regulatory network with inputs from LIF-BMP4 and 2i/3i media with concentrations*LIF*=85**and ***I***_***3=6***_**respectively.** Variation of residence time in the ES state. **(A)** Time series of NANOG (red) and OCT4-SOX2 (blue) when in LIF-BMP4 medium show significant fluctuations of NANOG expression between high and low levels. **(B)** Time series of NANOG and OCT4-SOX2 when in 2i/3i medium. **(C)** NANOG and OCT4-SOX2 distributions when in LIF-BMP4 medium. NANOG exhibits a wide distribution. **(D)** NANOG and OCT4-SOX2 distributions when in 2i/3i medium. **(E)** Time series showing the differentiation process occurring when the cells are maintained in LIF-BMP4 medium; NANOG (red), OCT4-SOX2 (blue) and G (green). The up-regulation of the differentiation gene G leads to an irreversible down-regulation of OCT4-SOX2 and NANOG. **(F)** The mean time that a stem cell remains in the ESC state increases with LIF concentration in the LIF-BMP4 stem cell medium.

In Figure 2A, we show the time series of OCT4-SOX2 and NANOG concentrations for a stochastic simulation of Equation 1 with *LIF*=85 for the parameters in Table 1. Not shown are the G and FGF4 time series; G fluctuates around extremely low levels and FGF4 is similar to OCT4-SOX2. Although OCT4-SOX2 remains at a fairly high level, NANOG displays a larger fluctuation. The corresponding distributions obtained from several Monte-Carlo runs (Figure 2C), show a tail for low NANOG levels with a peak at higher levels. OCT4-SOX2 displays less heterogeneity. This recapitulates the observed NANOG heterogeneity [18-20]. NANOG regulation occurs due to the competition between OCT4-SOX2, which directly induces NANOG, and suppression by FGF4, which itself is induced by OCT4-SOX2. This type of regulation implements an incoherent feed-forward loop [38]. It is the delay between the noisy OCT4-SOX2 induction of NANOG and its subsequent suppression through induction of FGF4, which itself is fluctuating, that creates the excess fluctuations observed for NANOG.

**Figure 3:**
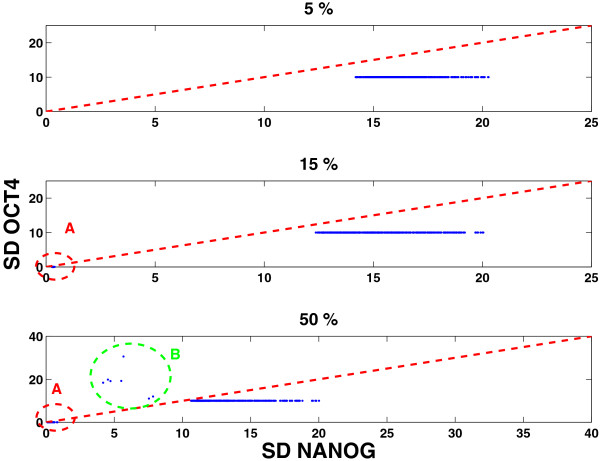
**NANOG and OCT4 standard deviations for multiple parameter sets.** Each parameter set was generated by randomly sampling ±ߙ5%, 15% and 50% around each parameter in Table 1 for *LIF*=100. In each case the NANOG standard deviation (SD) was greater than the OCT4-SOX2 SD, thereby suggesting that NANOG displays more heterogeneity. The oval A correspond to those parameter sets representing the differentiated state in which G is high and all other components are suppressed (hence low values of NANOG and OCT4). The points enclosed by the oval B represent parameter sets in which: NANOG is weakly regulated by itself; strongly suppressed by FGF4 and G. In addition OCT4 is weakly suppressed by G, which allows NANOG and G both to be expressed.

It has been shown that NANOG expression fluctuations reaching very low levels lead to irreversible commitment [18,24]. Hence we have built into our model the possibility of leaving the stem cell state by NANOG interactions with the differentiation gene G. Figure 2E shows NANOG fluctuations from a typical simulation. Should the NANOG expression hit a low level, G is relieved from the suppressive effects of NANOG, and is turned ON. Then G shuts OFF OCT4-SOX2 and hence the pluripotent state is transformed into differentiated one. Before this transition occurs, OCT4-SOX2 is at high levels but NANOG could be either high or low. It is only when NANOG reaches a very low level, through several consecutive degrading events in NANOG or OCT4-SOX2, and/or coupled with increase in FGF4 or G, that the switch to a differentiated state occurs. The above results which suggest the role of increased heterogeneity in NANOG as responsible for the fate of the stem cell, were obtained for the parameter set displayed in Table 1. To show that these results are robust to changes in parameter values we computed the fluctuations in NANOG and compared them with the fluctuations in OCT4, using the Linear Noise Approximation (LNA) [46-48] for a wide range of parameter sets (see Methods). In Figure 3, in each panel, we see the distribution of NANOG and OCT4 fluctuations for random parameter sets, for changes in parameters in increasing order (5*%*,15*%* and 50% respectively). For each distribution in parameter space, in the majority of the cases, we see that the highest fluctuations occur in NANOG expression. However, there are cases marked by the oval *A*, in the middle and last subplots, where NANOG and OCT4 fluctuations are extremely low. These represent those cases where the state of the cell is in the differentiated state, and hence the fluctuations in *G* would be highest. In the last subplot, the oval *B* represents those cases where the parameter sets corresponded to: weak NANOG auto-regulation, strong suppression of NANOG by FGF4, G and weak suppression of OCT4 by G. This therefore resulted in higher noise in OCT4 than NANOG, since the latter was strongly suppressed, at the same time G was unable to fully turn OCT4 off. However, the above results indicate that NANOG in most cases experiences the highest fluctuations for a wide range of parameters, thereby supporting its role as the “gatekeeper” of the stem cell state.

The pluripotent state has high levels of OCT4-SOX2 which are less heterogeneous than those of NANOG. The continuum of NANOG values spans both, high and low NANOG values. In [28] the NANOG distribution resulted in a bimodal one, due to a bistable switch-like mechanism. A critical point is that NANOG functions as a gatekeeper regardless of its exact distribution – at low values it is unable to repress G and hence causes a transition. Our simulations are consistent with the experimental observation that although LIF-BMP4 maintains ESCs, a low amount of differentiated cells are nevertheless produced. Simulations also show that increasing LIF improves the maintenance of stem cell cultures, i.e. the mean time that a cell, which is initialized as a stem cell, remains a stem cell increases with LIF value (see Figure 2F).

#### Stochastic dynamics under 2i/3i conditions

Recently, it was shown that ESCs can be maintained in 2i/3i [25] media, with the interesting result that heterogeneity in NANOG is lost. Our model assumes that the effect of small molecules in the 2i/3i medium is to suppress FGF4. This would relieve the suppression on NANOG. As it is shown in Additional file 1: Figure S1B, the system now exhibits a higher level of NANOG.

Figure 2B and Figure 2D show time series and distributions of OCT4-SOX2 and NANOG concentrations under 2i/3i conditions. They both fluctuate at high levels, with lower NANOG heterogeneity. Although LIF is not present, we assume that the stem cell state was initialized with G low and NANOG and OCT4-SOX2 high. Suppression of FGF4 leads to higher induction of NANOG and hence increased positive feedback between OCT4-SOX2 and NANOG through G, which ensures that their levels remain high. Hence, our simulations agree with the experimental observation of loss of NANOG heterogeneity with cells cultured in 2i/3i media. In Additional file 2: Figure S2 we display the mean and standard deviation of NANOG fluctuations using the LNA (see Methods). The figure shows that increasing 2i/3i while increasing the mean levels of NANOG decrease its fluctuations, while still being higher than fluctuations in OCT4. As mentioned earlier, although OCT4-SOX2 maintains pluripotency, it also induces FGF4, which pushes cells to differentiate. However, since FGF4 receptor signaling and GSK3 are inhibited in 2i/3i media, NANOG is not repressed (see Figure 1) and hence the NANOG high state is observed. This would be the true “ground state”, which requires no feeders or serum, except the small molecule inhibitors, which prevent differentiation as well as provide improved bio-synthetic environment for cell growth.

### Reprogramming – transition from somatic to iPS cells

Ectopic expression of the pluripotency transcription factors OCT4, SOX2, KLF4 enables the transition from somatic cells to iPS cells (ES like cells) [49]. One notes that NANOG is not required for reprogramming despite its hub role in the architecture. Reprogramming with only these three factors is inefficient. Hence, substantial efforts are made to overcome this drawback. Initially, over-expression of c-MYC was used but the addition of this factor increased not only the efficiency of reprogramming but also the tumorigenicity of the cells [49]. Recently, it has been shown that c-MYC can be replaced by GLIS1, which does not have the same tumorigenic effect [29]. However, the reprogramming process still remains inefficient and more understanding of the process on the molecular level is needed.

Our minimal dynamical model elucidates the reprogramming process when only OCT4, SOX2 and KLF4 are over-expressed and identifies the obstacles to overcome: The differentiation gene G antagonizes OCT4 and NANOG, and since it feeds back positively upon itself, once ON, it ensures that OCT4-SOX2 and NANOG are OFF. When OCT4-SOX2 is OFF, NANOG cannot be induced since OCT4 is unable to fulfill its epigenetic role of exposing the NANOG promoter for transcription. Hence, NANOG stays OFF. NANOG is also repressed by FGF4, which in this case would be low, since its inducer OCT4-SOX2 is OFF. Hence, over-expression of OCT4 is the key.

#### Deterministic analysis

**Figure 4:**
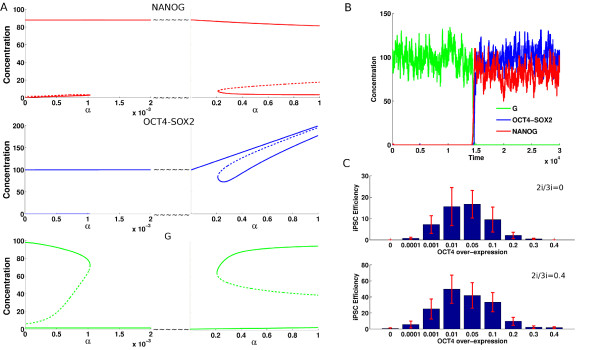
**Steady state analysis when OCT4-SOX2 over-expression is varied.** Time series concentrations of NANOG (red), OCT4-SOX2 (blue) and G (green) obtained from stochastic simulation when reprogramming occurs. Reprogramming efficiency when over-expression of OCT4-SOX2 is varied. **(A)**The steady state values of NANOG, OCT4-SOX2 and G as functions of over-expression (*α*) of OCT4-SOX2 for *LIF*=100 and *I*_3_=10 respectively. The gene regulatory circuitry is initially in the somatic state with G high and NANOG and OCT4-SOX2 low. The reprogramming occurs when G turns OFF while NANOG and OCT4-SOX2 turn ON (at *α*≃0.001). At higher values of *α*(*α*≃0.2), there now exist two states of the system: the existing ES state and the differentiated state which occurs since G turns ON while OCT4-SOX2 and NANOG switch to lower levels. **(B)** Time series of NANOG, OCT4-SOX2 and G for one case when the reprogramming was successful, G turns OFF while NANOG and OCT4-SOX2 turn ON for *LIF*=100 and *I*_3_ = 0.05 respectively. **(C)** The reprogramming efficiency for values of *α* varying from 0 to 0.4 and *I*_3_ taking 0 and 0.4 values, *LIF*=100

The parameter *α*in Equation 1 governs the OCT4 over-expression. In Figure 4A, the bifurcation diagram shows how the network components change with increasing *α*keeping the other parameters fixed. Three regions can be identified, approximately given by:

(i) *α*< 0.001, (ii) 0.001 <*α*> 0.2, (iii) *α*> 0.2. When going from (i) to (ii) OCT4 exposes NANOG and the high value of LIF (KLF4 over-expression [50,51]) induces NANOG, raising its expression to a sufficiently high level. The antagonism between NANOG and G, reduces G to low levels. This removes the G repression of OCT4-SOX2, which further induces NANOG. This implements the positive feedback loop, which makes the transition from the somatic state to the stem cell state. We note that with OCT4-SOX2 at high levels, FGF4 is induced, which does repress NANOG. However, this suppression is not strong enough to counter the induction from *LIF*, to switch it OFF. In addition, with 2i/3i media present, this effect can be significantly reduced.

As OCT4-SOX2 over-expression is further increased and reaches *α*≃0.2 (iii) another bifurcation occurs. Now, a new state emerges – a differentiated state in which NANOG can be at low levels while G is high. This new state is possible because over-expression of OCT4 leads to a relatively slow increase in G, as it can also be induced by OCT4 in the model (it is slow because it is also suppressed by NANOG which itself is strongly induced by OCT4). Once OCT4 crosses a certain threshold, G can overcome NANOG, leading to a differentiated state.

Our deterministic model analysis indicates that the stem cell circuit activation must be conditioned by OCT4-SOX2 over-expression. It is known that over-expression of OCT4 is mandatory for obtaining iPS cells in the laboratory [29]. However, too large an over-expression leads to a differentiated state [52,53]. Hence OCT4 has to be within a set of thresholds to achieve reprogramming [30]. It should be pointed out though that the latter result was obtained for human ESC.

#### Stochastic simulations

In Figure 4B we present the results of a corresponding stochastic simulation when the reprogramming was successful. Initially, the differentiation gene G has high expression whereas NANOG and OCT4-SOX2 have low expression values – the circuit is in the somatic state. Over-expression of OCT4-SOX2 with an optimal dosage *α*≃0.05 ensures that NANOG is strongly activated by OCT4 and hence the stem cell circuit switches to a stem cell state.

A major challenge is to increase the efficiency of the reprogramming process. We used our model to study the variation of reprogramming efficiency when the value of OCT4-SOX2 over-expression (*α*) is varied. Figure 4C shows the success rates obtained from multiple Monte Carlo simulations where the value of *α* was varied in the [0,0.4] range. Our results suggest that the dosage of reprogramming transcription factors has an important impact on reprogramming efficiency. This has been experimentally shown in a study on optimization of iPS cells generation [30]. We identified an optimal range for OCT4 added expression value (*α*) to be [0.001,0.1]. Such a success interval should be considered when reprogramming is conducted under LIF and BMP4 medium conditions. If the values of reprogramming factor expression is lower than the values in the reprogramming success range then reprogramming does not take place as OCT4 is not significantly expressed and it does not strongly induce NANOG. If the values of OCT4 are too high then they would correspond to values that lead to endoderm differentiation and reprogramming is not successful. Figure 4C shows a 10-fold decrease of the percentage of obtained iPS cells for values of *α*outside success intervals.

Our results show that the stem cell medium where the somatic cells are maintained after transduction also plays an important role in reprogramming efficiency. When reprogramming is successful, the differentiation gene G is OFF while NANOG and OCT4-SOX2 are at high values. The latter induces FGF4 which represses NANOG. The NANOG suppression by FGF4 influences negatively the reprogramming outcome. Thus, repression of FGF4 should have a positive impact on reprogramming efficiency. Indeed, when increasing 2i/3i concentration, an increase in iPS cell generation efficiency is observed (see Figure 4C). The percentage of iPS cells in this study represents the outcome from a minimalistic model and there must be additional factors not considered here which might modify the percentages. However, such factors would equally influence every scenario under consideration.

Nevertheless, our results demonstrate that setting the degrees of over-expression and choosing the iPS cell medium should be considered for optimizing reprogramming efficiency.

For completeness we performed similar analyses for a modified network topology without the differentiation gene G (see Additional file 3: Figure S3 and Additional file 4).

## Conclusions

Our computational model of the transcriptional dynamics of the embryonic stem cell suggests mechanisms in the simplified network feedback structure which allow cells to make a stochastic decision to exit from a stem cell state to a differentiated one. Such an event is random and occurs due to the internal noise of network components. In particular, we explicitly showed how NANOG heterogeneity enables such transitions. NANOG integrates several noisy signals. OCT4 both directly activates NANOG, as well as suppresses it through FGF4. When NANOG falls below a certain threshold, G gets activated, leading to shutdown of NANOG and OCT4. FGF4 can be suppressed by the 2i/3i media which leads to reduction of NANOG heterogeneity (specifically at higher levels) and hence to stability of the stem cell state, i.e the “ground state”. Our model could explain how the absence of the 2i/3i media, can result in the experimentally observed “leakage” to differentiated cells even under ideal culture conditions, since stochastic transitions of NANOG to relatively low levels can occur, in this case. The spontaneous commitment picture emerging from our model studies is consistent with the “permissive” scenarios suggested in the context of hematopoiesis [43,44]. One might speculate that this ESC property allows cells to form tissues in the natural environment of the embryo through a low rate of regulated differentiation events. Finally we studied the reprogramming scenario of somatic cells due to OCT4 over-expression. Our model was able to explain why reprogramming efficiency is biphasic with respect to OCT4 levels. Once reprogramming occurs, the external stimuli provide optimal conditions to maintain for the stem cell state.

Our simplified model could be expanded as more links in this network are explored. For example, recent work [54,55] suggests that NANOG is epigenetically modified by Ezh2, and as discussed in [56], this could have interesting consequences for a model seeking to describe NANOG fluctuations. It is expected that future experiments will discover additional network componentsand external media implicated to govern stem cell fate and reprogramming, which could be included into our current model. We have not explored the consequences of external noise due to the environmental signals: LIF and 2i/3i, which will be explored in another work. One area of immediate interest is to immerse our single cell stochastic dynamics in a spatial context of growing and dividing cells [57] with the aim to understand how noise in gene expression couples with mechanics and cell fate in the living embryo.

## Methods

### Model ingredients

NANOG is induced by OCT4-SOX2.

NANOG dimerizes and regulates itself positively [58].

The differentiation gene G positively auto-regulates itself and is repressed by NANOG.

LIF induces NANOG, presumably through Klf4 via the Stat3 pathway.

G is induced by OCT4-SOX2 and suppresses both NANOG and OCT4-SOX2.

Even though FGF4 is a growth factor, it is induced by OCT4-SOX2 [36]. Furthermore, FGF4 signaling was shown to suppress NANOG.

The 2i/3i medium has the effect of suppressing FGF4.

### Network dynamics

For the circuit in Figure 1, we obtain the following set of differential equations from a thermodynamic approach [59-63]. The equations describe the behavior of NANOG, OCT4-SOX2, FGF4 and differentiation gene G (SOX17), with concentration levels denoted by [N], [OS], [FGF] and [G]. The concentrations of LIF and small molecules in the 2i/3i medium are denoted as *LIF* and *I*_3_respectively.

(1) $$\begin{array}{lll} \frac{d[N]}{\mathrm{dt}}\;\; & =\;\;\frac{k_{0}[\mathrm{OS}](c_{0}+c_{1}{\left[N\right]}^{2}+k_{0}[\mathrm{OS}]+c_{2}\mathrm{LIF})}{\left(1+\left(k_{0}[\mathrm{OS}]\left(c_{1}{\left[N\right]}^{2}+k_{0}[\mathrm{OS}]+c_{2}\mathrm{LIF}+c_{3}{\left[\mathrm{FGF}\right]}^{2}\right)\right)+c_{4}[\mathrm{OS}]{\left[G\right]}^{2}\right)}\; & \;\\ \;\; & -\;\;\gamma [N],\; & \;\\ \frac{d[\mathrm{OS}]}{\mathrm{dt}}\;\; & =\;\;\alpha +\frac{\left(e_{0}+e_{1}[\mathrm{OS}]\right)}{\left(1+e_{1}[\mathrm{OS}]+e_{2}{\left[G\right]}^{2}\right)}-\gamma [\mathrm{OS}],\;\\ \frac{d[\mathrm{FGF}]}{\mathrm{dt}}\;\; & =\;\;\frac{\left(a_{0}+a_{1}[\mathrm{OS}]\right)}{\left(1+a_{1}[\mathrm{OS}]+a_{2}I_{3}\right)}-\gamma [\mathrm{FGF}],\; & \;\\ \frac{d[G]}{\mathrm{dt}}\;\; & =\;\;\frac{\left(b_{0}+b_{1}{\left[G\right]}^{2}+b_{3}[\mathrm{OS}]\right)}{\left(1+b_{1}{\left[G\right]}^{2}+b_{2}{\left[N\right]}^{2}+b_{3}[\mathrm{OS}]\right)}-\gamma [G],\; & \; \end{array}$$

In [37], an epigenetic effect was implicated by which OCT4 regulates the NANOG region by regulating the histone demethylases Jmjd2c. Here we implement such an effect for NANOG by assuming that that all TF’s which can bind to the Nanog promoter, do so only when the OCT4-SOX2 heterodimer [OS] is first bound to it. This functional form is motivated by the need to have OCT4-SOX2 make NANOG available for transcription. The parameter values used for the simulations are displayed in Table 1. Using the above deterministic equations we can obtain their steady state values as a function of the parameters. We also use the reaction rates from Equation 1 to write down a master equation, which has been simulated using the Gillespie algorithm to obtain the results in Figures 2 and 4.

We have performed Linear Noise Approximation analysis to prove the robustness of our results, as described below.

### Robustness analysis for NANOG fluctuations using the LNA

A second order expansion of the master equation, obtained from the transition rates in Equation 1, is called as the linear noise approximation (LNA) [46-48]. The assumption is that at steady state each network component fluctuates about its mean level, given by solving Equation 1, and is described by a Gaussian distribution. The fluctuations are described by a covariance matrix *C*. The diagonal components of *C*, describe the variances in each component, and the off diagonal components describe the cross correlations between the various species fluctuations. *C* is obtained at steady state by solving the Lyapunov equation given by,

(2) $$\mathrm{JC}+CJ^{T}+D\;\;=\;\;0,$$

where *J* is the Jacobian matrix, and *D* the effective diffusion matrix, which is obtained from Equation 1. We compute *C*, for a given parameter set and obtain the standard deviations for NANOG, OCT4 etc. This is then repeated for 500 randomly generated parameter sets. Each randomly generated parameter set is obtained by varying each of the parameters within a uniform distribution around the fiducial parameter set in Table 1 by ±5%, 15% and 50%. We did not vary the common degradation parameter *γ*, since the major effect would only lead to rescaling of time.

The bifurcation analysis was performed using JDesigner [64], Oscill8 [65]. Matlab (The Mathworks) was used to solve the differential equations, stochastic Gillespie simulations and the covariances of the network components using the LNA.

## Competing interests

The authors declare that they have no competing interests.

## Authors’ contributions

VC, VO, CP conceived and developed the project. VC, VO developed the simulation and analysis programs. VC, VO, CP analyzed the data and wrote the manuscript. All authors read and approved the final manuscript.

## Supplementary Material

Additional file 1 Figure S1. Steady state analysis of the stem cell circuit as functions of LIF and _*I*3_concentrations. The dashed lines indicate unstable states. **(A)** The steady state values of NANOG, OCT4-SOX2 and G as functions of LIF concentration. The plots display two states of the cell. (i) the stem cell state – high OCT4-SOX2/NANOG and low G and (ii) the differentiated state with low OCT4-SOX2/NANOG and high G **(B)** Similar graphs for small molecule differentiation inhibitors _*I*3_ concentration (here for 100). Although there are still two states, NANOG is still higher here than in the previous case. Shown also is the level of FGF4 (magenta), which is at ≃20, which is much lower than in the previous case. Click here for file

Additional file 2 Figure S2. NANOG mean and standard deviation as a function of the concentration of 2i/3i (*LIF*=0) computed using the LNA. In the upper panel, the mean value of NANOG , and the lower panel shows the standard deviation of NANOG. Also indicated as a red dashed line is the standard deviation of OCT4 which does not vary with 2i/3i. Click here for file

Additional file 3 Figure S3. Time series of [N] and [OS] for the stochastic dynamics of the alternative architecture with no differentiation gene G for *LIF*=100. A differentiation event; NANOG (red) and OCT4-SOX2 (blue). The OCT4-SOX2 expression is **not** lost in a differentiated cell in the model without G. Click here for file

Additional file 4 An architecture with no differentiation gene G. Click here for file
