# Supplementary material for: Probing the role of stochasticity in a model of the embryonic stem cell – heterogeneous gene expression and reprogramming efficiency
Source: BMC Syst Biol. 2012 Aug 13;6:98. doi: 10.1186/1752-0509-6-98 (PMC3468383; doi:10.1186/1752-0509-6-98)
Supplement: Additional file 4 — An architecture with no differentiation gene G. [file 1752-0509-6-98-S4.pdf]

Additional file 4 – An architecture with no differentiation gene G

Vijay Chickarmane[2], Victor Olariu[1] and Carsten Peterson*[1,3]

[1]Computational Biology & Biological Physics, Lund University, Lund, Sweden
[2]Division of Biology, California Institute of Technology, Pasadena, USA
[3]Lund Strategic Research Center for Stem Cell Biology and Cell Therapy, Lund University, Lund, Sweden

Email: Vijay Chickarmane - vchickar@caltech.edu; Victor Olariu - victor.olariu@thep.lu.se; Carsten Peterson *- carsten@thep.lu.se;

*Corresponding author

We performed a similar analysis for a modified network topology without the differentiation gene G. The corresponding dynamical equations are given by Equation 3.

$$\begin{aligned}
\frac{d[N]}{dt} &= \frac{k_0[OS](c_0 + c_1[N]^2 + k_0[OS] + c_2 LIF)}{(1 + (k_0[OS](c_1[N]^2 + k_0[OS] + c_2 LIF + c_3[FGF]^2))} \\
&- \gamma[N], \\
\frac{d[OS]}{dt} &= \alpha + \frac{(e_0 + e_1[OS])}{(1 + e_1[OS])} - \gamma[OS] \\
\frac{d[FGF]}{dt} &= \frac{(a_0 + a_1[OS])}{(1 + a_1[OS] + a_2 I_3)} - \gamma[FGF]
\end{aligned}$$

This model with no G can reproduce the time series expressions for the case with the cells in the ES state (Figure 2 A, B, C and D). However, and not surprisingly, when differentiation occurs, the OCT4-SOX2 expression is not lost since G is not present in the model to antagonize OCT4-SOX2 (Additional file 3: Figure S3). Hence, a model without G is not capable to cover the important ESC process of differentiation. When the same model with no G is applied to the reprogramming study it could reproduce the results in Figure 4B. However, this simplified model allows reprogramming even without over-expression of any of the transcription factors in the network. These result contradicts experimental findings where over-expression is necessary for successful reprogramming.
